# Banked Human Milk and Quantitative Risk Assessment of *Bacillus cereus* Infection in Premature Infants: A Simulation Study

**DOI:** 10.1155/2019/6348281

**Published:** 2019-02-03

**Authors:** Antoine Lewin, Gilles Delage, France Bernier, Marc Germain

**Affiliations:** ^1^Medical Affairs and Innovation, Héma-Québec, Montreal, QC, Canada; ^2^Department of Obstetrics and Gynecology, University of Sherbrooke, Sherbrooke, QC, Canada; ^3^Quality and Regulatory Affairs, Héma-Québec, Montreal, QC, Canada; ^4^Medical Affairs and Innovation, Héma-Québec, Québec, QC, Canada

## Abstract

**Background:**

Banked human milk (BHM) offers potential health benefits to premature babies. BHM is pasteurized to mitigate infectious risks, but pasteurization is ineffective against sporulating bacteria such as *Bacillus cereus*. Sepsis related to *Bacillus cereus* in premature infants is severe and can often be fatal. Even if a causal link has never been established, BHM has been suggested as a potential source of infection in premature infants.

**Objective:**

Our aim was to estimate the potential risk of *Bacillus cereus* infection in preterm infants caused by the ingestion of contaminated pasteurized BHM using different post-pasteurization release criteria (i.e., 9 sampling of 100 microliters versus the HMBANA guideline of 1 sampling of 100 microliters per pool).

**Methods:**

In the absence of scientific evidence regarding the risk of *Bacillus cereus* infection by the ingestion of BHM in premature infants, risk assessment using Monte Carlo simulation with the exponential dose-response model was performed. Three scenarios of infectious risk (annual incidence rate of 0.01%, 0.13%, and 0.2%) with 18 variations of the *B. cereus* virulent dose (from 0.5 CFU/ml to 200 CFU/ml) were simulated.

**Results:**

The mean risk differential between the two methods of post-pasteurization bacteriological control for realistic infectious doses of 30 to 200 CFU/ml ranges from 0.036 to 0.0054, 0.47 to 0.070, and 0.72 to 0.11 per million servings, for each of the three scenarios.

**Conclusion:**

Simulation highlights the very small risk of *Bacillus cereus* infection following the ingestion of pasteurized BHM, even in the worst case scenarios, and suggests that a 100-microliter sample for post-pasteurization culture is sufficient.

## 1. Introduction

Human milk is the preferred feeding for infants because of its known long-term benefits, such as an improvement of neurocognitive development [1–3], reduced risk for obesity [4], and protection of cardiovascular health outcomes [5]. There are some clinical settings in which human milk is particularly advantageous. This is the case of premature children who are at risk of necrotizing enterocolitis (NEC), a severe and sometimes fatal complication. Formula-fed infants are reported to have 6 to 20 times the risk of experiencing NEC compared with breast milk-fed infants [6]. Clearly, premature infants should be fed with their own mother's milk, whenever possible. However, banked human milk (BHM) is the best alternative feeding for preterm infants when maternal milk is not available or is insufficient [7, 8]. One possible exception that has been raised by some is the risk of systemic infection by *Bacillus cereus* through BHM [9, 10].

*Bacillus cereus* is a motile spore-forming, Gram-positive rod, aerobic, or facultative anaerobic bacteria, of the family Bacillaceae, that is ubiquitous worldwide in the environment (air, dust, and water) [11]. As a human pathogen, the organism is known for its role as a mediator of self-limited foodborne illness. However, *Bacillus cereus* is also known as a potential pathogen associated with severe local and systemic infections among immunosuppressed patients [12]. Cases in premature infants are quite rare, but neonatal sepsis related to *Bacillus cereus* is particularly severe and can lead to death [13–15]. Because sporulated form of this bacterium can resist the pasteurization process, which is the method used by most milk banks to safeguard against possible contamination, some researchers have raised the possibility that donated milk is a possible source of contamination [9, 13]. To our knowledge, the risk is purely theoretical since there has never been a single case of *Bacillus cereus* infection in a preterm infant proven to be caused by BHM ingestion.

**Figure 1:**
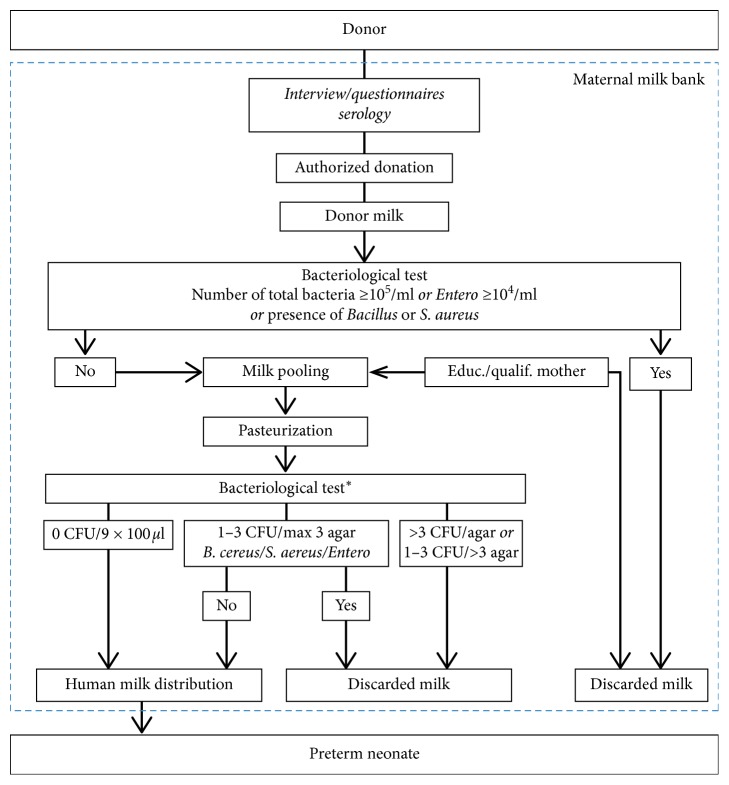
Human milk process at Héma-Québec milk bank. Bacterial control is based on 9 samples of 100 microliters per pasteurized milk pool. ^*∗*^Post-bacteriological sampling was done plating 100 *µ*l on 9 blood agar plates per pool. Results were categorized into three scenarios. First, if there is no observed bacteriological growth in the 9 samples, then pool milk is accepted for distribution. Second, if on a maximum of 3 plates bacteriological growth was found with no more than 3 CFUs per plate, other than *B. cereus*, *S. aureus*, or enterobacteria, then milk pool is accepted for distribution; if one of the above mentioned bacteria was found within the three positive plates, then pool milk was discarded. Third, if bacteriological growth was observed on more than 3 plates or if more than 3 CFUs were observed on a single plate whatever the bacterial species, then milk pool was discarded.

Practices vary between milk banks concerning milk pool volume, post-pasteurization routine bacterial assessment, bacteriological detection thresholds, and concentration standards for qualification [16]. Nevertheless, the large majority of North American milk banks assess post-pasteurization bacteriological control based on the Human Milk Banking Association of North America (HMBANA) guideline (i.e., one sample of 100 microliters per pool) [17]. According to this guideline, any bacteriological growth is unacceptable after the milk has been pasteurized. In the province of Quebec, Canada (total population: 8 millions), human milk bank post-pasteurization microbiological analyses of pooled milk are routinely made using 9 samples of 100 microliters per batch, which is nine times larger than that recommended by the HMBANA. The pasteurized milk is tested by inoculating 100 *µ*l of the lot that was pasteurized on 9 blood agar plates. Plates used for the bacterial detection are incubated 48 hours at 35 ± 2°C. Based on the results of this bacteriological assessment, a batch showing the presence of *Bacillus cereus*, regardless of the bacterial load, does not qualify for distribution (Figure 1). Pre-pasteurization microbiological testing was done for *B. cereus*, *S. aureus*, enterobacteria and total count screening for women entering the program. Women having the presence of *B. cereus* or *S. aureus* or enterobacteria at more than 10,000 CFU/ml, or a total count of more than 100,000 CFU/ml have their milk rejected and are contacted by a nurse to identify and correct their hygiene deficiencies. Historically, between 25 and 35% of the milk batches produced by our milk bank have been disqualified because of microbial contamination; unsurprisingly, *Bacillus cereus* was the microorganism found in 80–90% of the batches that were disqualified. We therefore considered the possibility of aligning our practice with the HMBANA's recommended 100 microliters of sample per batch. The question however was raised as to whether the risk of having a smaller sampling volume could reduce the sensitivity of our culture method to a point that it would lead to an unacceptable level of risk of *B. cereus* infection. Hence, the risk assessment is presented in this paper.

## 2. Materials and Methods

In the absence of scientific evidence regarding the risk of *Bacillus cereus* infection caused by the ingestion of BHM in premature infants, Monte Carlo stochastic simulation, reflecting variability and uncertainty of parameters, was used. Our risk evaluation followed the quantitative microbiological risk assessment (QMRA) method and therefore undertook consecutive steps, such as (1) hazard identification (described in the introduction section), (2) exposure assessment, (3) dose-response model, and (4) risk characterization [18]. Statistical analyses were performed using SAS 9.4 software (SAS Institute Inc., Cary, NC, USA).

### 2.1. Exposure Assessment

The aim of the exposure assessment section was to estimate the contamination concentration of *Bacillus cereus* in pasteurized pooled milk accepted after bacteriological assessment and consumed by preterm infants. The model was based on modular process risk (MPR) methodology [19, 20], as this chain divides naturally into a series of modules (i.e., pathways) which can be described by component processes (contamination, mixing, control, and detection). Figure 1 describes the donor milk chain model at Héma-Québec.

The assumptions and inputs underlying our exposure assessment model were as follows:

(1) *Pooled Milk Volume.* 40,000 milk donations were simulated to yield a total of 10,000 milk pools based on observed internal data, where (i) pooled milk volume production during the year 2017 at Héma-Québec was standardized, with a mean volume of 9,500 ml and SD 400 ml, and (ii) an average of 4 different milk donors are necessary to constitute one milk pool (given a mean milk volume per donation of 2,375 ml and SD 250 ml).
(2) *Pathogen Distribution.* The total number of pathogens per pasteurized milk pool (*T*) simulated was based on the hypothesis that (i) the prevalence of some level of contamination in any milk donation is 100%, given the ubiquity of *Bacillus cereus* in the environment, and (ii) pasteurization (*ε*) has no efficacy on reducing the concentration of *Bacillus cereus* contamination in a contaminated pool. Therefore, all simulated post-pasteurized milk pools were considered contaminated, and the rejection rate did not depend on the presence or absence of *Bacillus cereus* but on the sensitivity of the bacteriological control used. Contamination concentration per donation was simulated using lognormal distribution of mean 4.2 CFU/ml and SD 213. This simulation was based on our observed internal data (unpublished) with bacteriological post-pasteurization control using 9 samples of 100 microliters per pool: around 30% of pools with a mean *Bacillus cereus* concentration of 13 CFU/ml were detected and rejected. Moreover, this simulated concentration is consistent with the rejection rate reported in the literature where when using 1 sample of 100 microliters for post-pasteurization bacteriological control: around 10% of pools with *Bacillus cereus* were detected and rejected [21]:
  (1) $$\begin{array}{c} T_{\text{pool}}=\overset{Nc}{\underset{i=1}{\sum }}\left(1-\;\;\varepsilon \right)\left(V_{\text{d}}\times C_{\text{d}}\right), \end{array}$$
  where *T*_pool_ represents the total number of pathogens per pasteurized milk pool, *Nc* corresponds to the number of donations contaminated with *Bacillus cereus* per milk pool, and *Nc* is equal to the total number of contaminated donations. Because the prevalence of *Bacillus cereus* in milk donations was assumed to be 100%, all donations were assumed to be contaminated. *V*_d_ and *C*_d_ represent milk volume and *B. cereus* concentration per donation, respectively. The effect of pasteurization (*ε*) was set to zero as explained earlier.

(3) *Contamination Detection and Pasteurized Milk Pool Rejection.* Exposition of premature infants to *Bacillus cereus* depends on the probability of bacterial detection, given the sensitivity of the bacteriological control. Assuming that bacteria are randomly distributed in the pool, following a Poisson distribution, the probability of detection using 100 microliters was defined as follows:
  (2) $$\begin{array}{c} P_{\text{detec}}=1-e^{-\lambda x}, \end{array}$$
  where *λ* corresponds to the total CFU simulated by pool, and *x* corresponds to the volume of sampling per pool. Then, the number of rejected pools depending on the number of post-pasteurization bacteriological sampling tests (*k* = 1 or 9) follows a binomial distribution:

(3) $$\begin{array}{c} N_{\text{Rejec}}=\text{binomial}\left(P_{\text{detec}},\;k\right). \end{array}$$

(4) *Feedings.* The distribution of donor milk bank serving per premature infant was determined using internal reports [22]. The mean serving size was then estimated to be 100 ml.

### 2.2. Dose-Response Model

Because of the lack of information regarding the relationship between the dose ingested and infection following the ingestion of banked milk contaminated by *Bacillus cereus*, a full-risk assessment had to be modeled. The probability of infection resulting from the consumption of servings contaminated with *Bacillus cereus* (*P*) was based on an exponential dose-response model because it allows inferring the dose-effect relationship based on certain epidemiological hypotheses:

(4) $$\begin{array}{c} P=1-\;\;e^{-RN_{\text{D}}}, \end{array}$$

where *N*_D_ represents the total number of *B. cereus* in the serving and *R* is a constant specific to each pathogenic agent that expresses the probability of being infected by a single pathogenic microorganism present in the serving depending on the virulence of the bacteria [23–27]. In other words, the *R*-value is the probability of interaction between the host and the microorganism [28]. By rearranging equation (4), *R* can be expressed as follows:

(5) $$\begin{array}{c} R=\frac{-\left[\ln\left(1-P\right)\right]}{N_{\text{D}}}. \end{array}$$

The constant *R* was estimated using the approach described by Lindqvist and Westöö [25], in which the validity of the model depends on certain epidemiological assumptions. In the present study, because an exponential dose-response model is a nonthreshold model and since the simulation assumes that all serving are contaminated, all premature infants fed with banked milk are considered at risk. The total number of preterm children exposed to BHM per year and the mean number of servings per infant were estimated using internal distribution reports: approximately 1000 children were fed with milk supplied by the Héma-Québec human milk bank, with a mean of 40 portions per year.

In the absence of evidence regarding the incidence of *Bacillus cereus* infection in preterm infants fed with banked milk and the potential infectious dose of *B. cereus* in pasteurized milk, we cannot use an independent estimate of the constant *R*; therefore, variation in parameters for the risk characterization was made.

### 2.3. Risk Characterization

Three scenarios were built to estimate the annual incidence of illness in preterm infants caused by the ingestion of *B. cereus*-contaminated banked milk. As previously mentioned, there is no evidence of a causal link between banked milk contamination and *B. cereus* infection in preterm infants; we therefore had to make some epidemiological assumptions for the various scenarios being considered. As a factual basis for these scenarios, we used data from the largest observed outbreak of *Bacillus cereus* infections in newborns in Quebec (unpublished). In 2013–2014 at one hospital in our service area, 7 preterm infants became infected. An investigation concluded that these infections were likely to have been due to airborne contamination that resulted from construction work near the neonatal unit of the hospital. However, for the purpose of the simulation, we postulated that all these infections had been acquired by uncultured contaminated human milk (a worst-case situation). It should be noted that only one of these seven infants had been exposed to banked human milk.

Moreover, to build the three scenarios, we considered the following denominator data: (1) the total number of births in Quebec during that year (*n* = 88,867), (2) the total number of births less than 2.5 kg in Quebec (*n* = 6,464), and (3) the total number of births at the hospital where the *B. cereus* infections occurred (*n* = 3,500). Depending on the denominator being taken, this resulted in annual estimated incidence rates of 0.01%, 0.13%, and 0.2% for scenarios 1, 2, and 3, respectively. For each scenario, in order to estimate the uncertainty of the constant *R* and because no information concerning the minimal infectious dose of *Bacillus cereus* in human milk is available, sensitivity analyses were performed. Eighteen variations of the infectious dose of *Bacillus cereus* in human milk were simulated, from 0.5 CFU/ml to 200 CFU/ml. Therefore, *R-*value ranged from 1.2*E* − 10 to 5.00*E* − 8, 1.62*E* − 9 to 6.50*E* − 7, and 2.50*E* − 9 to 1.00*E* − 7 for scenarios 1, 2, and 3, respectively (Table 1).

## 3. Results

Ten thousand pools, each constituting of 4 mothers, were simulated with a mean volume of 9.5 liters of SD 0.4 and a mean *Bacillus cereus* contamination concentration of 4.05 CFU/ml, SD 103 and geometric mean of 0.16.

### 3.1. Detection and Rejection Probability

Following our simulation, using post-pasteurization bacterial control sampling of 100 microliters per milk pool, 9,026 of the 10,000 simulated pools containing *Bacillus cereus* would not be detected and would be in the milk bank inventory for distribution. The average concentration of *Bacillus cereus* contamination in the pools qualified for distribution would be 0.64 CFU/ml. In addition, a rejection rate of 9.74% (974 pools) would be observed, with an average *Bacillus cereus* contamination concentration of 35.74 CFU/ml. This rejection rate is similar to those reported by human milk banks that use a post-pasteurization sample of 100 microliters for culture [21, 29]. If post-pasteurization bacterial control sampling consisted of nine sample of 100 microliters per milk pool, 6,996 pools with a mean *Bacillus cereus* concentration of 0.21 would not be detected and would be qualified for distribution. 3,004 pools would have been detected with an average *Bacillus cereus* concentration of 13.01 CFU/ml and a rejection rate of 30% (Table 2). This simulated rejection rate and the concentration in the rejected pool are consistent with those of our internal observations.

In summary, concentration of *Bacillus cereus* in pools put into the inventory would be higher by 0.43 CFU/ml average when detected using one sample of 100 microliters versus 9 samples of 100 microliters. Moreover, the rejected milk volume difference would be 19,312 liters, corresponding to 20.3% of the total volume simulated.

### 3.2. Residual Risk Differential

**Figure 2:**
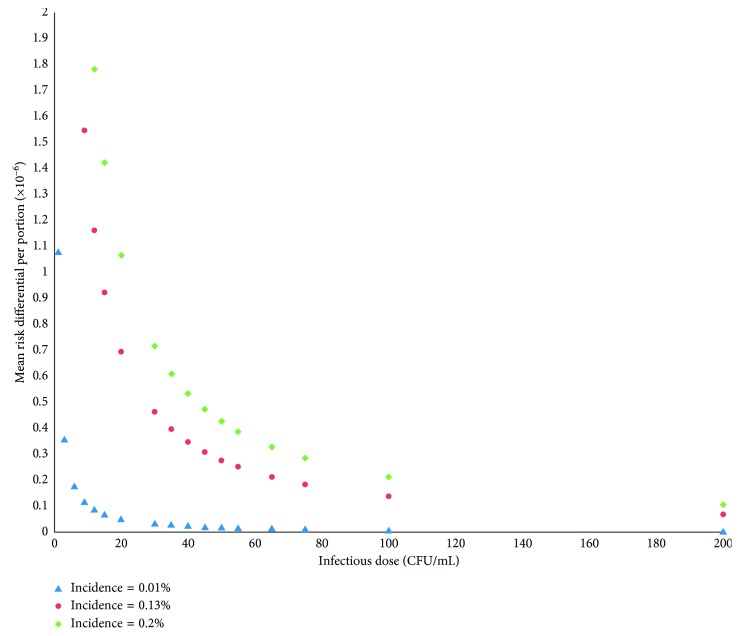
Mean risk differential per portion using the two different post-pasteurization bacteriological control criteria. Horizontal axis corresponds to the 18 variations of the infectious doses according to the 3 different incidence scenarios identified by the corresponding colors. The vertical axis corresponds to the mean risk differential per million portions.

Figure 2 represents the mean risk differential per million between the two methods of post-pasteurization bacterial control (i.e., 1 sample versus 9 samples of 100 microliters) by scenario and infectious dose. This figure shows that for the vast majority of scenarios, even among the most pessimistic, the mean risk differential associated with sampling of 1 test rather than 9 tests of 100 microliters is well below the threshold of 1: 1 million additional infections per serving. It is only when we suppose that the minimal infectious dose is 20 CFU/ml or less that we observe a risk difference greater than 1: 1 million.

Table 3 presents detailed data concerning the residual risk scenarios comparing a 100-microliter sample to a 900-microliter sample for post-pasteurization culture when varying the infectious dose per serving. The mean risk differential for scenario 1 ranges from 2.15 to 0.00539 per million for virulent dose of 0.5 to 200 CFU/ml. For scenarios 2 and 3, the mean risk differential ranges from 27.8 to 0.070 per million and from 42.8 to 0.108, respectively.

## 4. Discussion

Proven invasive infections due to *Bacillus cereus* have been reported among immunosuppressed patients, particularly preterm neonates, but given the scarcity of cases, the pathophysiology of these infections is not well known. Moreover, assessment of the origin of contamination due to organisms such as *B. cereus* that are widely disseminated in the environment is often difficult and may not yield an obvious source [11]. Nonetheless, in outbreak investigations, contamination through respiratory route was often documented [11, 15]. Several studies have also assessed the putative role of human milk in *B. cereus* infection in premature infants; however, clinical strains of *B. cereus* never matched the strains found in milk, and therefore, the tracking of the origin of *B. cereus* to contaminated bank milk was, to date, never demonstrated [6, 9].

Our analysis cannot make any statement regarding the actual risk of *Bacillus cereus* infection resulting from the ingestion of contaminated banked human milk. Even if the risk is real, which remains unproven yet, it is likely to be extremely small, based on the various scenarios that we explored in this simulation. The question of whether there is a real threat posed by *Bacillus cereus* to the safety of banked human milk therefore remains unresolved.

What our simulation shows is that, even in very pessimistic scenarios regarding a causal link between banked milk and *B. cereus* infection, a more stringent culture-sampling strategy makes very little difference in the risk incurred by exposed infants. At the same time, because of the ubiquitous nature of this bacterium, increasing the sampling volume and thus the sensitivity of the culture method can lead to much higher rates of product disqualification. More specifically, our analysis shows that the risk difference per serving associated with a sampling of 1 instead of 9 tests of 100 microliters is well below 1: 1 million infections, a risk that can be considered negligible. In all scenarios, it is only when the minimal infectious dose was 20 CFU/ml or less for a 100 ml serving that the risk differential was greater than 1 infection/1 million portions; such minimal infectious doses are clearly unrealistic for *Bacillus cereus*. Also, average concentrations of *Bacillus cereus* found in the qualified milk batches would only be slightly increased, from 0.21 to 0.64 CFU/ml, which remains well below the standards applied to formula milk (i.e., 50 CFU/g in Europe and 100 CFU/g in the USA, Australia, and New Zealand) [30–32]. By comparison, the literature on human and animal exposure by inhalation to different doses of spores of *Bacillus anthracis* reports a minimal infectious dose of at least 600 CFU [33, 34]. If we assume the same minimal infectious dose of 600 CFU per serving for *B. cereus* in BHM, this would still be ten times higher than the average *B. cereus* concentration remaining in the qualified milk pools. Furthermore, because of the much lower potential for the pathogenicity of *Bacillus cereus* compared with *B. anthracis* [33, 34], this hypothesis is extremely aggressive. Finally, the gain in supply resulting from a less-stringent culture method, and its potential impact on product availability for the prevention of necrotizing enterocolitis, largely exceeds the very low theoretical differential risk as calculated by these models [32, 35, 36]. In our operational setting, changing the sampling volume from 900 to 100 microliters is expected to decrease the rate of disqualified, culture-positive pools from 30 to 10 percent. This will represent a major improvement in our capacity to meet the demand for premature infants.

To our knowledge, this is the very first study, using Monte Carlo simulation, which estimates the potential risk of *Bacillus cereus* infection in preterm infants fed with banked human milk. The major limitation of our study is that we had to estimate many of the parameters used in the absence of empirical data concerning their value. Also, the simulation was based on the premise that any bacterial load in the milk pool is homogeneously distributed, which is probably the case in the vast majority of pools in our experience. However, the possibility of this load being distributed unevenly due to an inadequate milk mixing process in rare instances cannot be ruled out.

## 5. Conclusion

Our simulation results suggest that, if it exists at all, the risk of *Bacillus cereus* infection following the ingestion of pasteurized banked milk is extremely small. Our analysis also shows that even in worst-case scenarios, a 100-microliter sample for post-pasteurization culture is amply sufficient to mitigate this risk; a larger sampling volume would only lead to a higher rate of disqualification for this important health-care resource, without having any significant positive impact on safety.

Nevertheless, despite no specific mention about *Bacillus cereus* contamination in the majority of the guidelines, this simulation highlights the importance of the combined use of pasteurization and bacteriological investigation before human milk distribution for extremely preterm babies. In addition, future studies investigating the association between *Bacillus cereus* concentration and infection of premature infants are needed.

## Figures and Tables

**Table 1:** *R*-value for the exponential dose-response model based on simulated scenarios and variation of the infectious dose.^*∗*^

Dose^1^	Total CFU per serving	*R* scenario 1	*R* scenario 2	*R* scenario 3
200	20,000	1.2*E* − 10	1.62*E* − 9	2.50*E* − 9
100	10,000	2.5*E* − 10	3.25*E* − 9	5.00*E* − 9
75	7,500	3.3*E* − 10	4.33*E* − 9	6.66*E* − 9
65	6,500	3.8*E* − 10	5.00*E* − 9	7.69*E* − 9
55	5,500	4.5*E* − 10	5.91*E* − 9	9.09*E* − 9
50	5,000	5.0*E* − 10	6.50*E* − 9	1.00*E* − 8
45	4,500	5.5*E* − 10	7.22*E* − 9	1.11*E* − 8
40	4,000	6.2*E* − 10	8.13*E* − 9	1.25*E* − 8
35	3,500	7.1*E* − 10	9.29*E* − 9	1.42*E* − 8
30	3,000	8.3*E* − 10	1.08*E* − 8	1.67*E* − 8
20	2,000	1.25*E* − 9	1.63*E* − 8	2.50*E* − 8
15	1,500	1.67*E* − 9	2.17*E* − 8	3.33*E* − 8
12	1,200	2.08*E* − 9	2.71*E* − 8	4.16*E* − 8
9	900	2.78*E* − 9	3.61*E* − 8	5.56*E* − 8
6	600	4.17*E* − 9	5.42*E* − 8	8.33*E* − 8
3	300	8.33*E* − 9	1.08*E* − 7	1.67*E* − 7
1	100	2.50*E* − 8	3.25*E* − 7	5.00*E* − 7
0.5	50	5.00*E* − 8	6.50*E* − 7	1.00*E* − 7

^*∗*^Annual incidence of infection of 0.01%, 0.13%, and 0.2% for scenarios 1, 2, and 3, respectively. ^1^Dose in CFU/ml.

**Table 2:** Average contamination of *Bacillus cereus* in the accepted and rejected pools.

Variables	*N*	%	Volume, mean (in l)	Concentration, mean (SD)
Lots not detected 1 sampling of 100 *µ*l	9,026	90.3	85,747.82	0.64 (1.68)
Lots not detected 9 samplings of 100 *µ*l	6,996	70.0	66,435.46	0.21 (0.36)

Lots detected 1 sampling of 100 *µ*l	974	9.7	9,254.59	35.74 (329.11)
Lots detected 9 sampling of 100 *µ*l	3,004	30.0	28,566.95	13.01 (188.01)

**Table 3:** Mean risk of infection by ingestion of banked maternal milk by serving according to different levels of *Bacillus cereus* infectious dose in premature neonates.^*∗*^

Infectious dose^1^	Scenario 1			Scenario 2			Scenario 3		
	100 *µ*l sampling	9 × 100 *µ*l sampling	Risk differential	100 *µ*l sampling	9 × 100 *µ*l sampling	Risk differential	100 *µ*l sampling	9 × 100 *µ*l sampling	Risk differential
200	7.99*E* − 9	2.60*E* − 9	5.39*E* − 9	1.03*E* − 7	3.34*E* − 8	6.96*E* − 8	1.59*E* − 7	5.14*E* − 8	1.08*E* − 7
100	1.60*E* − 8	5.19*E* − 9	1.08*E* − 8	2.06*E* − 7	6.69*E* − 8	1.39*E* − 7	3.17*E* − 7	1.03*E* − 7	2.14*E* − 7
75	2.13*E* − 8	6.93*E* − 9	1.44*E* − 8	2.75*E* − 7	8.92*E* − 8	1.86*E* − 7	4.23*E* − 7	1.37*E* − 7	2.86*E* − 7
65	2.46*E* − 8	7.99*E* − 9	1.66*E* − 8	3.17*E* − 7	1.03*E* − 7	2.14*E* − 7	4.88*E* − 7	1.58*E* − 7	3.3 *E* − 7
55	2.90*E* − 8	9.45*E* − 9	1.95*E* − 8	3.75*E* − 7	1.22*E* − 7	2.53*E* − 7	5.76*E* − 7	1.87*E* − 7	3.89*E* − 7
50	3.19*E* − 8	1.04*E* − 8	2.15*E* − 8	4.12*E* − 7	1.34*E* − 7	2.78*E* − 7	6.34*E* − 7	2.06*E* − 7	4.28*E* − 7
45	3.55*E* − 8	1.15*E* − 8	2.40*E* − 8	4.58*E* − 7	1.49*E* − 7	3.09*E* − 7	7.05*E* − 7	2.29*E* − 7	4.76*E* − 7
40	3.99*E* − 8	1.30*E* − 8	2.69*E* − 8	5.15*E* − 7	1.67*E* − 7	3.48*E* − 7	7.93*E* − 7	2.57*E* − 7	5.36*E* − 7
35	4.56*E* − 8	1.48*E* − 8	3.08*E* − 8	5.89*E* − 7	1.91*E* − 7	3.98*E* − 7	9.06*E* − 7	2.94*E* − 7	6.12*E* − 7
30	5.32*E* − 8	1.73*E* − 8	3.59*E* − 8	6.87*E* − 7	2.23*E* − 7	4.64*E* − 7	1.06*E* − 6	3.43*E* − 7	7.17*E* − 7
20	7.99*E* − 8	2.60*E* − 8	5.39*E* − 8	1.03*E* − 6	3.34*E* − 7	6.96*E* − 7	1.58*E* − 6	5.14*E* − 7	1.07*E* − 6
15	1.06*E* − 7	3.46*E* − 8	7.14*E* − 8	1.37*E* − 6	4.46*E* − 7	9.24*E* − 7	2.11*E* − 6	6.86*E* − 7	1.42*E* − 6
12	1.33*E* − 7	4.33*E* − 8	8.97*E* − 8	1.72*E* − 6	5.57*E* − 7	1.16*E* − 6	2.64*E* − 6	8.57*E* − 7	1.78*E* − 6
9	1.77*E* − 7	5.77*E* − 8	1.19*E* − 7	2.29*E* − 6	7.43*E* − 7	1.55*E* − 6	3.52*E* − 6	1.14*E* − 6	2.38*E* − 6
6	2.66*E* − 7	8.65*E* − 8	1.79*E* − 7	3.43*E* − 6	1.11*E* − 6	2.32*E* − 6	5.28*E* − 6	1.71*E* − 6	3.57*E* − 6
3	5.32*E* − 7	1.73*E* − 7	3.59*E* − 7	6.87*E* − 6	2.23*E* − 6	4.64*E* − 6	1.06*E* − 5	3.43*E* − 6	7.17*E* − 6
1	1.60*E* − 6	5.19*E* − 7	1.08*E* − 6	2.06*E* − 5	6.69*E* − 6	1.31*E* − 5	3.17*E* − 5	1.03*E* − 5	2.14*E* − 5
0.5	3.19*E* − 6	1.04*E* − 6	2.15*E* − 6	4.12*E* − 5	1.34*E* − 5	2.78*E* − 5	6.34*E* − 5	2.06*E* − 5	4.28*E* − 5

^*∗*^Simulation scenario was made after post-pasteurized microbiological control using detection of 100 microliters vs 9 × 100 microliters per pool and for an annual incidence of infection of 0.01%, 0.13%, and 0.2% corresponding to scenarios 1, 2, and 3, respectively. ^1^Infectious concentration in CFU/ml.

## Data Availability

The simulation code and the data used to support the findings of this study are available from the corresponding author upon request.
